# Tumor Microenvironment in Acute Myeloid Leukemia: Adjusting Niches

**DOI:** 10.3389/fimmu.2022.811144

**Published:** 2022-02-22

**Authors:** Thomas Menter, Alexandar Tzankov

**Affiliations:** Pathology, Institute of Medical Genetics and Pathology, University Hospital Basel, University of Basel, Basel, Switzerland

**Keywords:** AML, TME, bone marrow niches, bone marrow stromal cells, T-cells

## Abstract

Acute myeloid leukemias (AML) comprise a wide array of different entities, which have in common a rapid expansion of myeloid blast cells leading to displacement of normal hematopoietic cells and also disruption of the microenvironment in the bone marrow niches. Based on an insight into the complex cellular interactions in the bone marrow niches in non-neoplastic conditions in general, this review delineates the complex relationship between leukemic cells and reactive cells of the tumor microenvironment (TME) in AML. A special focus is directed on niche cells and various T-cell subsets as these also provide a potential therapeutic rationale considering e.g. immunomodulation. The TME of AML on the one hand plays a vital role for sustaining and promoting leukemogenesis but - on the other hand - it also has adverse effects on abnormal blasts developing into overt leukemia hindering their proliferation and potentially removing such cells. Thus, leukemic cells need to and develop strategies in order to manipulate the TME. Interference with those strategies might be of particular therapeutic potential since mechanisms of resistance related to tumor cell plasticity do not apply to it.

## Introduction

Acute myeloid leukemia (AML) can be defined as a clonal expansion of hematopoietic progenitor cells showing a stop of differentiation and maturation at various stages (1). AML has a severe impact on affected patients with a general five-year survival rate of 24%, a median survival of 8.5 months, and an even worse prognosis in the most commonly affected patient group of people over the age of 65 showing a median survival of only 2.7 months (2). This data and the fact that no increase in survival rates could be achieved in the last decades, show the urgent need for improved treatment modalities.

The mainstay of AML therapy includes intensive chemotherapy and allogeneic hematopoietic cell transplantation (AHCT) (3). The rationale behind AHCT is the idea that blasts remaining after chemotherapy will be eliminated by the transplanted donor-immune cells (graft-versus-leukemia effect). However, in a considerable fraction of patients, relapses occur. On the other hand, many – especially elderly – AML patients are not eligible for this therapeutic option (4) and, thus, other therapeutic strategies must be explored. Improved understanding of the biology of AML and especially its microenvironment, including the exploration of the applicability of chimeric antigen-receptor T cells (CAR-T), might open new treatment options and significantly improve the outcome of patients. A key to achieve this goal is a profound understanding of the bone marrow niche, which provides physical protection and release of pro-survival factors for leukemia cells (5, 6), and the interaction between AML blasts and the immune system as potential target for immunotherapeutic approaches (7, 8).

As hematopathologists, we are confronted daily with evaluation of bone marrow biopsies and the characterization of its cellular components. This work has resulted in the conceptualization of various own studies dealing with the TME of hematopoietic neoplasms, which will be presented in this review along with seminal publications of other groups. So the focus of this review is the TME of myeloid tumors, particularly AML, from the viewpoint of histopathology that may mechanistically explain how AML blasts interact with the TME to support growth and survival. For a detailed view on therapeutic approaches targeting the TME in AML, we refer to other excellent reviews focusing on that topic (9, 10).

## The Microenvironment of the Bone Marrow Niche

The term “bone marrow niche(s)” has been coined to describe the specialized interplay of various cells and soluble factors providing the basis for sufficient and well-regulated hematopoiesis from the hematopoietic stem cells (HSC).

The bone marrow consists of a delicate vascular architecture of arterioles, veins and specialized sinusoids that enable trafficking of cells and soluble factors to the blood stream and vice versa, particularly through the fenestrated basal lamina of the sinusoids (11). Another important component of the bone marrow niches are stromal cells, which to a large extent comprise the adipose tissue, a longtime neglected component (12). Many authors distinguish a perivascular and an endosteal niche (10). It is still a matter of debate whether this distinction really can be made, since there is cumulating evidence in the support of the existence only of the former (13).

Hematopoiesis occurs in a circadian fashion effectuated by sympathetic nerve fibers in the bone marrow (14), and HSC themselves have been shown to be subject to respective oscillation, which is orchestrated by the sympathetic nervous system (15). Signaling *via* β3-adrenergic receptors regulates nestin-expressing mesenchymal cells and their cell progenitors (MSC) (16), which are of core importance for the *perivascular bone marrow niches* (17). As a net effect, β3-adrenergic signaling e.g. regulates trafficking of HSC (18).

The *endosteal bone marrow niche* (17) has been described as a shelter for HSC, and its regulation is orchestrated by osteoblasts and their progenitors (19). Osteoblasts form protective layers for HSC, they can also keep them in a non-circulatory state *via* cell-cell adhesion molecules (20). This niche is preserved after treatment by chemotherapy and radiation, being the source of bone marrow renewal after such insults (21). As also capillaries seem to play an as important role in the endosteal niche as in the perivascular niche, it has been proposed to relinquish the separation between different niches. Just recently Panvini et al. presented human data on the presence of nestin+ capillary-like tubes (NCLTs), not surrounded by sub-endothelial perivascular cells in direct contact to the bone line and spatially correlated with hematopoietic stem/progenitor cells and possibly involved in regulating human hematopoiesis within the endosteal compartment (22). This group also showed that the endosteal niche capillary network gets destroyed in the course of AML evolvement in favor of the central perivascular niche.

The *perivascular niche* is considerably (about 9 times) larger. Its MSC can differentiate into osteoblasts, adipocytes, chondrocytes and fibroblasts (23), and are particularly important for HSC by secreting factors such as C-X-C motif chemokine 12 (CXCL12), stem cell factor (SCF) and interleukin (IL) 7 (16). Vice versa, MSC can be modulated by HSC to improve their own microenvironment (24). The most voluminous compound of the perivascular niche, the adipocytes of the bone marrow, have been shown to be different from adipocytes elsewhere in the body. Their amount steadily increases during lifetime and they are involved in HSC maintenance and proliferation by secreting adipokine and adiponectin (25), in addition of being an important source of nutrients for bone marrow cells. Production of CXCL12, IL3 and IL6 has also been documented for this cell type (26). Finally, the vascular partition consists of sinusoids and arterioles, which – based on single cell analysis – must be regarded as different subcompartments (27, 28), e.g. sinusoids being mainly involved in cell egression from the bone marrow, while arterioles play a central role in nutrient and oxygen supply.

Macrophages – as in many other forms of TME – also are a pivotal part of the bone marrow niches. They can give rise to osteoclasts, influence osteoblasts and CXCL12-secretion by MSC to maintain homeostasis of HSC (29). Interestingly, the progenies of HSC, megakaryocytes also play a backloop role for HSC by secreting several cytokines such as insulin-like growth factor 1, platelet factor 4 and transforming growth factor beta (TGFβ) (30).

## The Tumor Microenvironment of MDN and MPN: Similarities and Differences to AML

A weighty proportion of AML cases does not present as *de novo* disease but represents clinical or at least genetically perceptible evolution (31) of background clonal hematopoietic stem cell disorders such as myelodysplastic syndromes (MDS) that are now proposed to be called myelodysplastic neoplasms (MDN), or myeloproliferative neoplasms (MPN), the microenvironment of which has been the focus of research. MDN patients carry a high risk (up to 25%) of developing AML (32), while in MPN this risk is more variable, depending on the respective entity, and is below 10%. Interestingly in this regard, MDN cells can induce transformation of normal MSC into MSC with proinflammatory characteristics, which can then support leukemic progression (33), while vanishing nestin-positive niches is a well-established mechanism of MPN pathogenesis (34). Moreover, it has been shown in animal models that certain molecular dysfunctions in MSC can induce an MPN-like phenotype (35, 36). This just shows two brief insights into the interaction and relationship of neoplastic ells and their environment of these disease groups. In the following paragraphs when focusing on different cell subsets and soluble factors, we will figure out current knowledge in regard to the TME of AML.

## The Role of Mesenchymal Stem Cells and Their Progenies in AML

**Figure 1:**
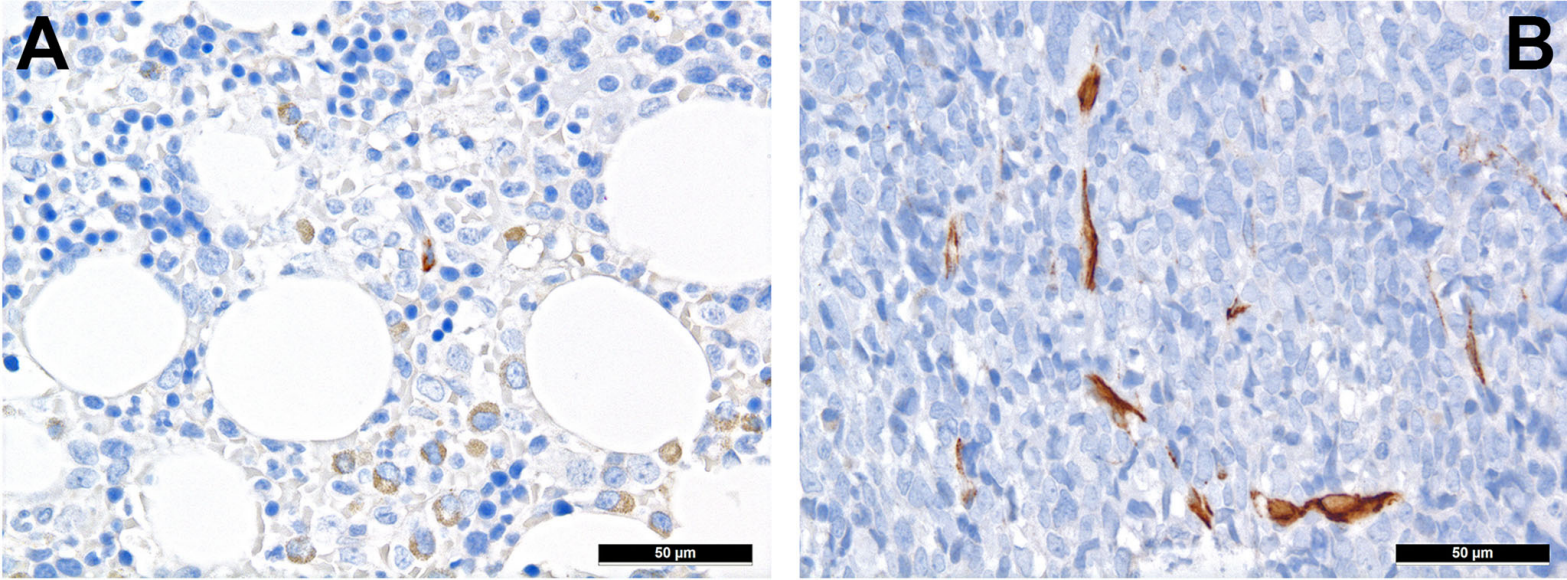
Distribution of nestin+ cells of the perivascular niches in AML. **(A)** Nestin expression in MSC-equivalents in normal bone marrow (immunohistochemistry, 400x); **(B)** Increased presence of nestin-positive MSC-equivalents in a case of AML suggesting importance of the former cells for AML survival (immunohistochemistry, 400x).

MSC are pluripotent cells that can differentiate into different types of mature cells as described above. During lifetime, there is a “physiological” change of MSC, which is reflected by increase of adipocytes and loss of osteoblasts (37). This alteration might also explain the well-known increasingly reduced bone marrow cellularity in the course of life. The underlying change of MSC differentiation is achieved *via* different mechanisms, including altered cytokine levels, resulting in decreased Wnt-related signaling and intensified RhoA-related signaling (38) as well as reduced Runx2 signaling, which is important for osteoblastic differentiation (39). “Senescence” that can be attributed to (cumulative effects of) pro-inflammatory cytokines, and changes of the sympathetic nerve system during life affect MSC (40). Indeed and as a cumulative result of altered signaling in cancer, nestin-positive MCS are significantly reduced in several hematologic malignancies. In a study on the TME of MPN, we could demonstrate that there is both a reduction of sympathetic nerve fibers and, consecutive, of nestin-expressing MSC in MPN, which is linked to the effects of proinflammatory cytokines that are increased in MPN, and can be to a part reverted by administering β3-adrenergic agonists (34). Interestingly, reduced numbers of nestin-positive MSC also correlate with another inflammatory condition, GvHD in AHCT patients (41). On the other hand, the role of nestin-positive MSC is different in AML as here, no depletion of these MSC has been observed (**Figures 1A, B**) and it is speculated that AML blasts are in need of MSC for survival and chemotherapy resistance, being dependent on the oxidative phosphorylation and tricarboxylic acid cycle activity, and antioxidant defense of the latter (42). Whether the genuine disruption of MSC niches in MPN (34) compared to rather increased MSC-niche densities MDN (43) may additionally explain the higher transformation rate into AML of the latter, is an intriguing hypothesis that remains to be resolved.

Analogously to what has been mentioned in the previous section discussing the relationship between MPN and their TME, mutations can also occur in the MSC themselves, which is documented in MPN linked to mutations in *Ptpn11* (44) or progression of AML fostered by *Ctnnb1* mutations in osteoblasts (45) in mouse models. This shows a new path of leukemogenesis, initiated not by mutations occurring in HSC but in MSC with secondary mutations occurring in the blasts. Similar to what is known from studies on bone marrow senescence, disruption of sympathetic nerve signaling also happens in AML. In mouse models on both AML and MPN, the destruction of Schwann cells and reduced sympathicotonus significantly altered bone marrow niches and promoted the increase of leukemic stem cells and disease progression (34, 46). Interestingly, β3-adrenergic agonists helped to restore physiological conditions and prevented disease progression.

AML blasts can push MSC to differentiate into osteoblasts, being as supportive for leukemia as for HSC in general (47), and - *via* the mechanism of leukemic stem cell protection - in the endosteal niche in particular (48). This seems to occur *via* secretion of proinflammatory cytokines such as CCL3 or thrombopoietin (TPO). Single cell RNA sequencing studies could already provide deeper insights into the reprogramming of MSC *in vivo*: Baryawno et al. elegantly demonstrated that different MSC are involved, including e.g. significant changes in osteoblast subsets and decreases differentiation into adipocytes, accompanied by upregulation of hypoxia-related genes in MSC (28).

Adipocytes are getting diminished by AML expansion in the bone marrow, which seems to be not only due to spatial disproportion, but also by preferential MSC-differentiation towards the osteoblastic lineage (49). On the other hand, adipocytes provide nurture to AML cells *via* release of fatty acids and also are important for bioavailability/unavailability of many lipophilic drugs (50), so their importance for AML cells is still difficult to interpret. Interestingly, the latter property of these cells is suspected being one reason for the inferior survival of elderly AML-patients as adipocyte-rich niches may provide a protective environment for AML blasts (51).

AML blast tightly interact with the endothelium. It has long been known that AML show an increased microvascular density compared to non-neoplastic bone marrow (52). Vascular endothelial growth factor (VEGF) is a major player in this relationship: besides fostering angiogenesis it can also inhibit apoptotic signaling in AML cells and support proliferation by upregulating secretion of GM-CSF by endothelial cells (53). Moreover, adhesion of leukemic stem cells through E-selectin to the vascular niche protects the former from the lethal effect of chemotherapy, which can be specifically counteracted (54).

**Figure 2:**
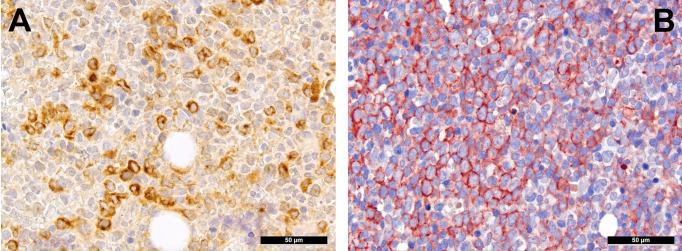
Expression of hyaluronic acid binding proteins in AML. **(A)** Diffuse expression of RHAMM in AML cells (immunohistochemistry, 400x); **(B)** Diffuse expression of CD44v6 in AML cells (immunohistochemistry, 400x).

Extracellular matrix components are an important factor for cell (im-)mobility and, thus, also a vital component of the TME. Already ten years ago, we could show the importance of RHAMM/CD168 (**Figure 2A**), the receptor for hyaluronic acid-mediated motility, as a negative prognostic marker for AML patients (55). CD44 that also interacts with hyaluronic acid (**Figure 2B**), is a type I transmembrane protein serving - especially in its sialofucosylated form - as adhesion molecule with ligands such as osteopontin, E- and L-selectin and fibronectin, and is an important factor for homing of HSC, and particularly of leukemic stem cells (56). Blocking CD44, the most commonly variant of which expressed in AML is v6 that is moreover linked to poor outcome, has been shown to prevent persistence of AML blasts in the bone marrow niche upon chemotherapy, which is otherwise linked to the quiescent-promoting- and, thus, cell-protective TME functions of CD44 (57).

## The Role of Lymphocytes and Monocytes in AML Immune Escape

As seen in many other tumors, immune escape is an important survival strategy for AML. AML blasts have been shown to impede the formation of immune synapses (58) and impair cytotoxic activity of T-cells (59). Generally, and in AML in particular, immune escape can be divided into different strategies including hiding of the malignant cells from the immune system up to manipulation of various immune-cell subsets.

**Figure 3:**
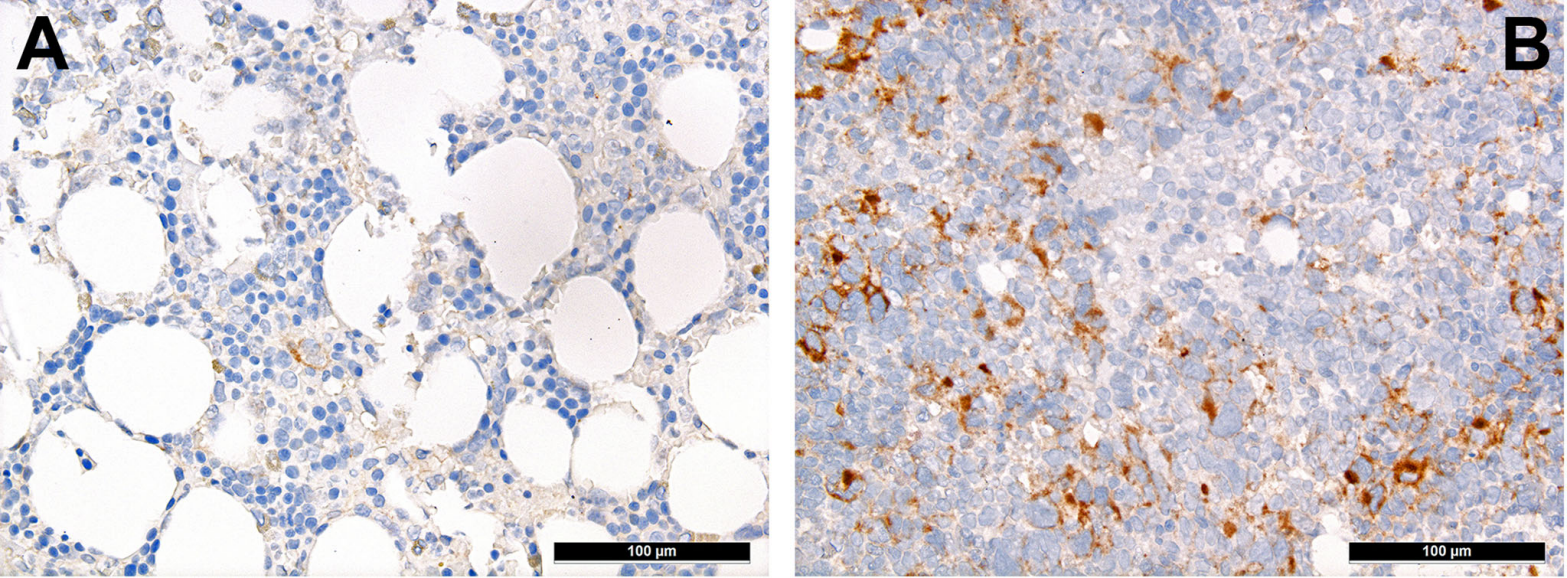
Distribution of PD-L1+ mononuclear tumor-infiltrating cells in AML. Case of relapsing AML showing high expression of PD-L1 **(B)** in contrast to very scarce expression of PD-L1 in the adjacent non-neoplastic bone marrow (**A**; immunohistochemistry, 200x).

**Figure 4:**
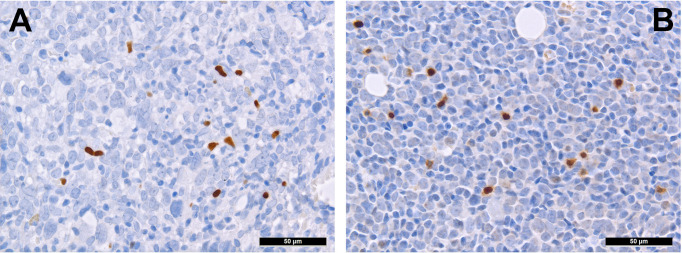
Distribution of regulatory T-cells and TH1-cells in AML. Presence of regulatory T-cells expressing FOXP3 **(A)** and Tbet **(B)** in bone marrow involved by AML (immunohistochemistry, 400x).

T-cells can be divided into regulatory and cytotoxic T-cell subgroups. Regulatory T-cells (Tregs) dampen inflammatory responses, both by secreting anti-inflammatory cytokines as well as silencing cytotoxic T-cells (60). Thus, Tregs’ effects to provide protection from the immune system may be important for AML. AML blasts express one of the most potent cell-contact immune silencers, programmed death ligand 1 (PD-L1; **Figures 3A, B**), which increases with disease progression (61). They can also produce reactive oxygen species (62) and indolamine-2, 3-dioxygenase (IDO), which both promote differentiation towards Tregs (63). Just recently, a study by Ragaini et al. demonstrated that an IDO1-related immune gene signature predicts overall survival in AML (64). Another target to induce T-cell anergy is T-cell immunoglobulin and mucin domain 3 (TIM3), which binds to and is activated by galectin-9, the latter being highly expressed on AML blasts, which leads to an activation of several downstream signaling pathways such as the MAPK/ERK, PI3K- and AKT (65). TIM3 can stimulate the production of IDO and thus foster immune evasion (66). Yet, regarding the role of TIM3-expression AML, there are still conflicting results (67–69). Secretion of inducible T-cell co-stimulator ligand (ICOSL) and IDO’s product, N-formylkynurenine, furthermore contribute to an immunosuppressive environment stimulating the expansion of Tregs, while limiting cytotoxic activity (70, 71). Low Treg levels have been generally associated with better outcomes in AML (72). However the very dynamic nature of these interaction can be appreciated from the following example: in a retrospective cohort we could show higher numbers of FoxP3+ Tregs (**Figure 4A**) in the early phases after induction therapy are associated with higher complete remission rates and better overall AML survival (73), contrasting their role when assessed at baseline AML biopsies (74). We hypothesized that this is attributed to active changes of the T-cell compartment reflecting the stereotypic recovery of the immune system after chemotherapy. Due to their immunomodulatory abilities, the expanding Tregs could foster the recovery of normal hematopoietic cells effectuated e.g. by limiting activity of effector T-cells, which may otherwise react to potential neo-epitopes unmasked due to cell destruction caused by chemotherapy. Accordingly, the beneficiary role of Tregs becomes evident when looking at lower rates of GvHD in Treg-high AHCT patients (75). Alongside, high expression levels of the surface molecules mentioned such as PD1/PD-L1 and TIM3 are associated with worse prognosis in AML, thus suggesting potential relevance for therapeutic interventions, with first immune checkpoint inhibition trials being underway (76) although – as alluded to above –investigations of the expression of these markers still produces conflicting results (69). Yet and in accordance with the described above, it seems that immune checkpoint inhibition as a single therapy is not successful, therefore currently combination therapies are being explored (77).

T-cell exhaustion can further be achieved by alterations of the metabolic microenvironment of AML, which can be characterized as glutamine-rich (78) and an arginine-deprived (79). Therefore, administering L-asparaginase (80) and inhibiting arginine deprival (81) have been both shown to be promising options for AML. Further immunomodulating drugs such as lenalidomide have also been tested in AML. In a translational side-project of a randomized clinical study (HOVON103 AML/SAKK 30/10), addressing the role of TME in AML treated by chemotherapy with or without addition of lenalidomide, we could show that its addition may be beneficial to (elderly) patients suffering from AML with multilineage dysplasia, where it led to a reduction of microvascularization and, likely, to an intensified specific T cell-driven anti-leukemic response (82). Lenalidomide promotes the degradation of two transcription factors, Aiolos and Ikaros, of the cereblon-mediating signaling (83, 84). Lenalidomide affects both neoplastic cells - by facilitating apoptosis, and the surrounding T-cells - by activating them *via* enhanced secretion of IL 2 (85). Indeed, under lenalidomide, the amount of T-bet+ T cells more consistently increased (82), which might be interpreted as a sign of increased T cell-driven immune response against the tumor cells. Consistently, this T-bet+ T-cell subgroup (**Figure 4B**) is a significant contributor to T-cell activation (86), and has a potential beneficiary effect on AML outcome as it fosters anti-AML immunity (87).

The role of B-cells has not been investigated at a larger scale so far. Cheng et al. investigated the prognostic importance of the TME in AML and showed a negative impact of memory B-cells (yet not statistically significant) on survival, while increased numbers of naïve B-cells had a positive impact (88).

Besides T-cells of the adaptive immune system, AML blasts also alter the function of innate immune system cells, namely NK cells and macrophages. AML blasts downregulate surface molecules needed for their recognition by NK cells *via* the receptor natural killer group 2 member D (NKG2D), and release altered NKG2D-ligands reducing the cytotoxic activity of NK cells (89, 90). Another mechanism to evade NK-cell recognition and, thus, destruction is inhibiting release of interferon γ (91). Similar to solid tumors, AML blasts induce a shift of macrophages towards M2 polarization, M2 macrophages being immunosuppressive, supporting angiogenesis and tissue repair (92). Elevated levels of M2 macrophages in AML patients have been described *in vivo* and in mouse models (93). Another population manipulated by AML blasts are myeloid-derived suppressor cells, which are derived from monocytes (94). These cells cause T-cell inactivity *via* several mechanisms ranging from PD-L1 expression to secretion of cytokines such as IL10 and/or TGFβ. In line with M2-macrophages and Tregs, these myeloid-derived suppressor cells are also more abundant in AML patients (94), and, consistently, they seem to be a risk factor for disease progression of MDN to overt AML (95). Finally, AML blasts also show a defective antigen-presentation by downregulation of human leukocyte antigens (HLAs) helping to render them invisible to immune cells (96).

These complex interactions between AML cells and non-neoplastic immunomodulating cells seems to represent a major source of difficulties for applying CAR-T in such instances (97), besides difficulties in identifying targets not also expressed by non-neoplastic HSC (98). As detailed above, induction of myeloid-derived suppressor cells and Tregs significantly reduces T-cell responses, which also holds true for CAR-T responses. Targeting CD33, which is not only expressed on AML blasts, but also on the myeloid-derived suppressor cells is a potential strategy to overcome this dilemma (99). Respecting AML-TME interactions, a phase I/II trial is currently running to explore the application of CD44v6 CAR-T in AML (https://clinicaltrials.gov/ct2/show/NCT04097301). Constructing CAR-T with costimulatory domains to dampen the influence of Tregs might be another potential approach (100). A further obstacle to the efficacy of CAR-T is the secretion of various soluble factors by AML blasts, which will be discussed in the next paragraph.

## Soluble Factors Affecting the TME

AML cells depend on numerous cytokines and soluble factors provided by cells of the bone marrow niches. In addition, several adhesion factors seem to be essential for AML cells within these niches.

**Figure 5:**
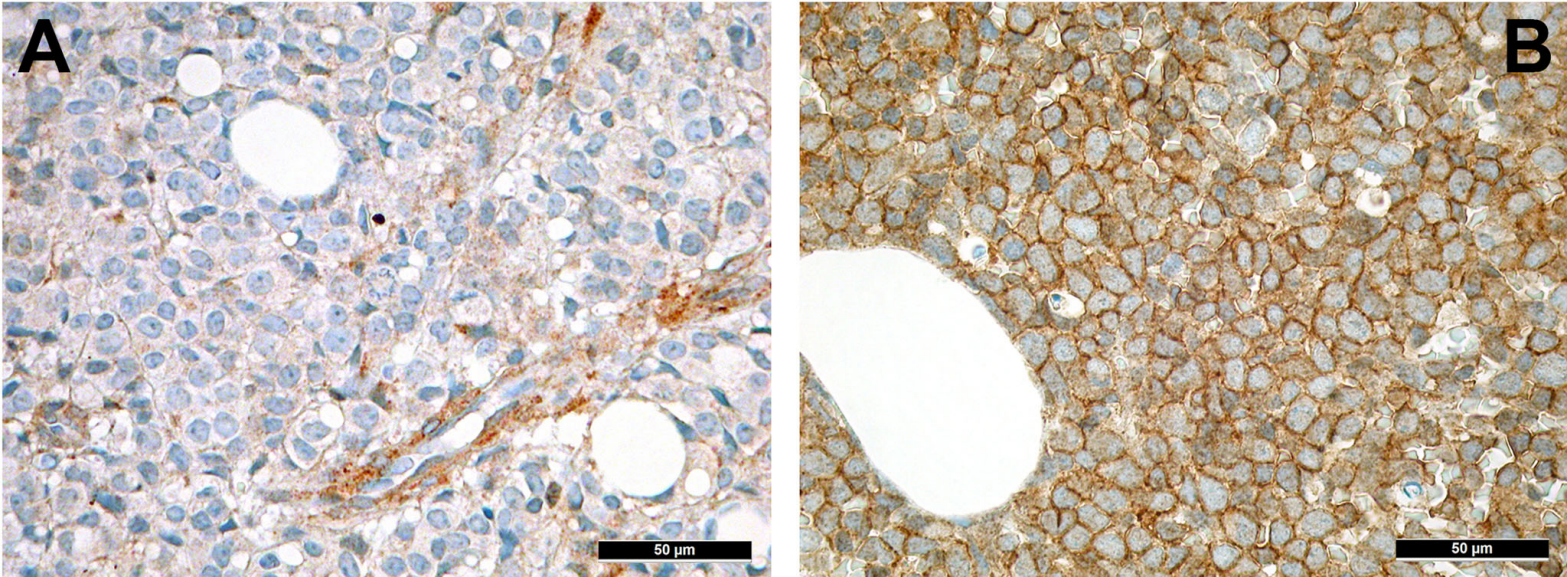
Expression of members of the CXCL12-CXCR4 axis in AML. **(A)** Expression of CXCL12 in perivascular MSC-equivalents in AML (immunohistochemistry, 400x); **(B)** Diffuse expression of the receptor of CXCL12, CXCR4, on AML blasts (immunohistochemistry, 400x).

CXCL12 (**Figure 5A**) is secreted by MSC under the orchestration of sympathetic nervous signaling, and regulates leukocyte trafficking as mentioned above (101). Interactions with its receptor, CXCR4, increase retention of HSC to the bone marrow niches and decreases their progenies’ migration into the bloodstream. Several years ago, we could show that active CXCR4 signaling is associated with an inferior prognosis in AML as a consequence of an increased retention to the bone marrow associated with an enhanced chemoresistance of leukemic cells (102). Indeed, under chemotherapy, AML cells often overexpress CXCR4 (**Figure 5B**) that facilitates overcoming apoptotic stimuli and improving cell survival by entering a state of quiescence and stromal protection, thus, being less amenable to the effects of cytotoxic drugs (103). CXCR4 antagonists such as plerixafor or the monoclonal antibody ulocuplumab are currently investigated in several clinical trials (104). CXCR4-antagonists mobilize leukemic stem cells into the blood stream and block several survival pathways as discussed above, thus rendering AML cells more chemo-susceptible. Importantly, granulocyte colony-stimulating factor (G-CSF) that is commonly given to support recovery from chemotherapy, seems to antagonize CXCR4 effects too, and is claimed to improve AML outcomes by means of this mechanisms, too (105).

ILs are an integral component for the regulation of the immune system. AML blasts can secrete a variety of ILs to adjust the TME to their needs: secretion of IL1β promotes growth of blasts, thus, serving as an autocrine growth factor, while suppressing non-neoplastic HSC (106) also by overturning adrenergic signaling in the perivascular niches (see above). By binding circulating IL2 *via* CD25, AML blasts can reduce T-cell activity (107). IL8 seems to be important for chemoresistance (108), and its secretion by endothelial cells is propagated by AML blasts. IL10 is secreted by MSC of the TME and correlated with worse survival in AML (109). By secreting IL10, MSC can induce immune tolerance, a well-established function of IL10 (110), and thereby shield AML blasts from detection by the immune system.

## Conclusions

**Figure 6:**
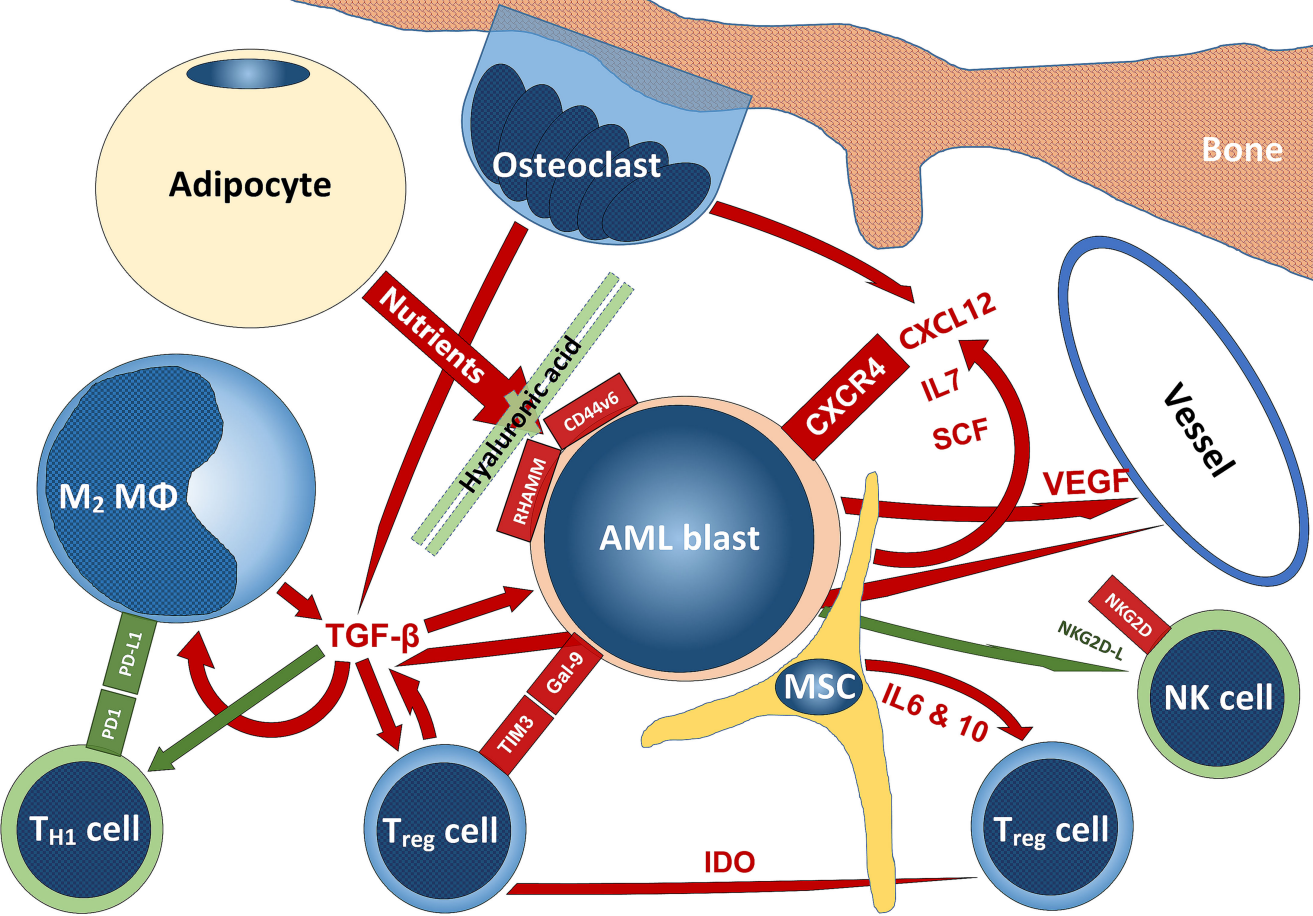
Schematic summarizing some interactions of AML with the TME. Stimulatory signaling is delineated in red, inhibitory – in green; tumor-promoting cells are blueish, tumor-suppressing cells – greenish, while niche-cells and nutrient-supplying cells are yellowish. The scheme does not claim to be complete and mainly reflects aspects that have been addressed in this review. For abbreviations, we kindly refer to the manuscript body.

In this review, we tried to give an overview of the massively expanding knowledge on the complex interactions of AML with their TME. The delicate structures and functions of the bone marrow niches are profoundly altered in order to help AML blasts to survive, expand and escape immune surveillance. Both, various cellular subsets as well as soluble factors play important roles in this settings (**Figure 6**). With more and more knowledge generated from treating solid tumors with immunotherapy, it is now also beginning to be explored in AML, especially in instances treated by chemotherapy. An area still not profoundly investigated is AML in the setting of AHCT. Indeed and as may be anticipated from the information on the interaction of AML with its TME reviewed here, increased knowledge on different pathways and mechanisms of action mediated through the TMA that may - if therapeutically adjusted - counterbalance graft-versus-host-, while supporting graft-versus-leukemia effects in the setting of AML treated by AHCT has the potential to considerably improve outcome. Further understanding and finding modes of action of immunotherapies and therapies targeting non-immunologic AML-TME interactions to counterbalance the manipulative effects of AML blasts on the bone marrow niche and its constituents has the potential to improve the prognosis of AML patients both at initial stages as well as in the relapse setting and achieving control in the minimal residual disease setting.

## Author Contributions

Both authors conceived and wrote the manuscript. All authors contributed to the article and approved the submitted version.

## Conflict of Interest

The authors declare that the research was conducted in the absence of any commercial or financial relationships that could be construed as a potential conflict of interest.

## Publisher’s Note

All claims expressed in this article are solely those of the authors and do not necessarily represent those of their affiliated organizations, or those of the publisher, the editors and the reviewers. Any product that may be evaluated in this article, or claim that may be made by its manufacturer, is not guaranteed or endorsed by the publisher.
